# Picosecond Lifetimes of Hydrogen Bonds in the Halide Perovskite CH_3_NH_3_PbBr_3_

**DOI:** 10.1021/acs.jpcc.4c04686

**Published:** 2024-11-26

**Authors:** Alejandro Garrote-Márquez, Lucas Lodeiro, Norge Cruz Hernández, Xia Liang, Aron Walsh, Eduardo Menéndez-Proupin

**Affiliations:** † Departamento de Física Aplicada I, Escuela Politécnica Superior, Universidad de Sevilla, Seville E-41011, Spain; ‡ Departamento de Química, Facultad de Ciencias, Universidad de Chile, Las Palmeras 3425, Ñuñoa 7800003, Santiago Chile; § Thomas Young Centre and Department of Materials, Imperial College London, London SW7 2AZ, U.K.

## Abstract

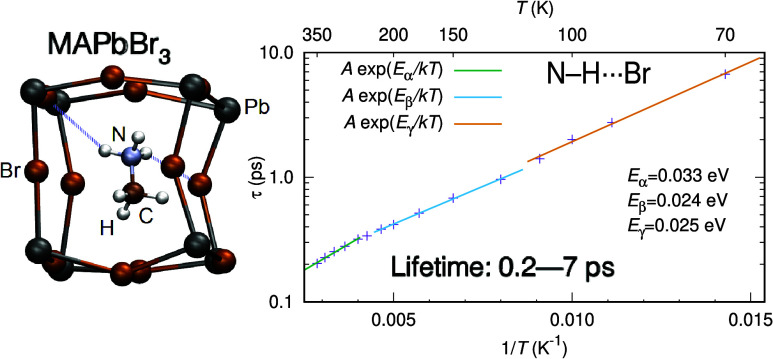

The structures and properties of organic–inorganic perovskites are influenced by the hydrogen bonding between the organic cations and the inorganic octahedral networks. This study explores the dynamics of hydrogen bonds in CH_3_NH_3_PbBr_3_ across a temperature range from 70 to 350 K, using molecular dynamics simulations with machine-learning force fields. The results indicate that the lifetime of hydrogen bonds decreases with increasing temperature from 7.6 ps (70 K) to 0.16 ps (350 K), exhibiting Arrhenius-type behavior. The geometric conditions for hydrogen bonding, which include bond lengths and angles, maintain consistency across the full temperature range. The relevance of hydrogen bonds for the vibrational states of the material is also evidenced through a detailed analysis of the vibrational power spectra, demonstrating their significant effect on the physical properties for this class of perovskites.

## 1 Introduction

Tin–lead halide perovskites, including hybrid organic–inorganic halide perovskites (HOIHP), have become important for use in optoelectronic devices such as solar cells and light-emitting diodes (LEDs), showing very high energy conversion efficiency.^1−6^ In the first publication on this field in 2009, Kojima et al.^7^ reported MAPbBr_3_ and MAPbI_3_ (MA = CH_3_NH_3_) based solar cells that showed high photovoltages of 0.96 and 0.61 V with external quantum conversion efficiencies of 65 and 45%, respectively.

In the subsequent years, researchers have made significant progress in improving the efficiency and performance of tin–lead halide perovskite solar cells.^8−12^ There are significant advances in the development of new synthesis and manufacturing methods, which allow to achieve greater stability and reproducibility.^13,14^ Research has also been conducted to understand and control the mechanisms of photogeneration. In terms of applications, solar cells based on tin–lead halide perovskites have demonstrated energy photoconversion efficiencies (PCE) over 26%, approaching the performance of crystalline silicon solar cells.^9,15,16^ In addition, efficient LEDs based on tin–lead halide perovskites, which can emit light in different colors, have been developed.^17−19^ However, despite the progress achieved so far, there are still technical and scientific challenges for industrial use, one of the main ones being the long-term stability of these materials.^20−23^

The structure of MAPbBr_3_ offers a combination of chemical and physical characteristics that can be exploited to enhance its long-term stability.^24−26^ Due to the composition and size of the ions (Pb^2+^) and bromine (Br^–^), it is possible to form a stable crystalline lattice. The relatively large size of the methylammonium ion in the structure helps to maintain the stable crystalline lattice and reduces the probability of structural degradation.^27^ Furthermore, lead halide perovskites like MAPbBr_3_ tend to exhibit good thermal and environmental stability compared to other perovskites as well as lower sensitivity to water and oxygen.^13,24^

**Figure 1:**
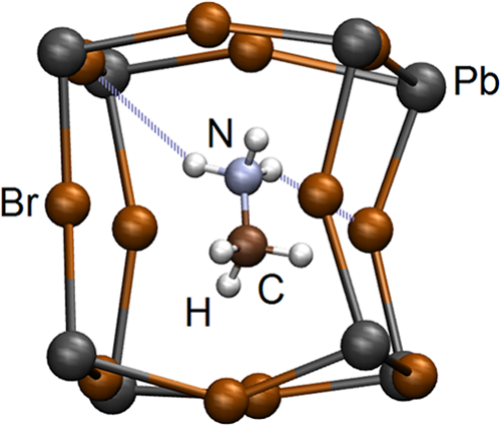
Representation of the MAPbBr_3_ perovskite structure. Hydrogen bonds are indicated by thin-dashed lines. Image created with VESTA.^28^

In the structure of the perovskite MAPbBr_3_, the lead ions (Pb^2+^) occupy the sites of a simple cubic lattice, and the bromine ions (Br^–^) occupy the positions between neighboring lead ions. Thus, each lead is bound to six bromines forming an octahedron with lead at the center and bromines at the corners, which connect adjacent octahedra. The methylammonium cations (CH_3_NH_3_^+^ or MA^+^) are found in the cavities formed between corner-sharing PbBr_6_ octahedra. Each MA^+^ cation is surrounded by 12 bromine and 8 lead ions, forming a cube around the methylammonium cation, as seen in Figure 1.

Temperature changes cause solid-state phase transitions in MAPbBr_3_.^29−31^ At low temperatures (<145 K), MAPbBr_3_ adopts an orthorhombic crystal structure. For temperatures in the range of 145–235 K, MAPbBr_3_ adopts a tetragonal structure, and for higher temperatures, the crystal symmetry becomes cubic. The latter is important for technological applications at room temperature. On the other hand, the stability and durability of hydrogen bonds (HBs) in lead halide perovskites are important since they play a role in the structure stability and dynamics, and hence the electrical, optical, and mechanical properties of these materials. In this way, there have been previous studies where the hydrogen bonding in perovskite solar cells (PSCs) are recently summarized, including each functional layer and interface.^32^ So, there are three notable aspects:

1. The stability of the HBs determines the general stability of tin–lead halide perovskites. If the HBs are weak and prone to breakage, the perovskite structure can become unstable and break down easily.^33^
2. The HBs modify the distortion of PbBr_6_ octahedra, which, in turn, determine the absorption and emission bands in the electromagnetic spectrum.
3. The electrical conductivity could be modified due to the mobility of ions under the influence of HBs,^34−36^ which can allow the development of materials with ionic transport properties.

The existence of HBs is based on certain characteristics of hydrogen and the atoms with which it can form bonds. These characteristics are defined by the International Union of Pure and Applied Chemistry (IUPAC)^37^ and are as follows:

1. Electronegativity difference: Hydrogen must be covalently bonded to a highly electronegative atom (X), such as oxygen (O), nitrogen (N), or fluorine (F). These atoms can attract the electrons of the bond toward them, generating a negative and positive partial charge on the electronegative and hydrogen atoms, respectively.
2. Bond length: The length of the donor-hydrogen covalent bond (X–H) usually gets larger upon formation of HB. This is due to the electrostatic interaction between the positive partial charge of the hydrogen and the negative partial charge of the acceptor atom (Y).
3. Bond angle: The HB angle is close to 180°, i.e., the atoms involved (X, H, Y) lie close to a straight line.
4. Forces of attraction: HBs are stronger than van der Waals forces but weaker than covalent or ionic bonds. This relatively strong electrostatic attraction results in characteristic properties of molecules containing them, such as higher boiling and melting points than expected for compounds of similar mass.

In MAPbBr_3_, two types of X–H···Br HBs are possible, with X = C or N. The role of carbon as the X donor is arguable due to the smaller electronegativity of carbon compared to oxygen, nitrogen, and fluorine. However, both nitrogen and carbon fulfil the current IUPAC criteria as HBs donors, and their occurrence in HOIHPs is also supported by noncovalent interaction (NCI) analysis.^38−41^ NCIs, including HBs, can be effectively identified using electronic structure calculations, particularly through the reduced density gradient method.^40^ Furthermore, Figures S1 and S2 in the Supporting Information show a small enlargement of the X–H bond length correlated with a decrease of the H···Br distance.

Lee et al.^41,42^ have performed a thorough analysis of the relationship between the MA cation orientation, the octahedral tilting, and the formation of N–H···I HBs in MAPbI_3_. Aided by density functional theory (DFT) calculations, they demonstrated a relationship between octahedral tilting and the frequency and strength of HBs in the orthorhombic and tetragonal phases of MAPbI_3_. They have also shown that octahedral tilting contributes to perovskite stabilization and that this stabilizing effect is comparatively greater in HOIHPs than in inorganic perovskites. For the tetragonal phase of MAPbI_3_, they showed that MA cations can have eight possible orientations of the C–N axis, which fall in two possible environments of MA in the Pb_8_I_12_ cuboctahedral cavity. These two environments show a significant energy difference (45 meV/MA) that is associated with HBs. Mondal and Mahadevan^43^ have extended the analysis to other HOIHPs, including chlorides and bromides, focusing on the influence of hydrogen bonds and the orientation of the organic cation within the inorganic network. In the specific case of orthorhombic MAPbBr_3_, they described different bonding patterns between hydrogen and bromine atoms, which generate variations in the Pb–Br–Pb bond angles and in the distance of the N–H···Br bonds. Other contributions are reviewed elsewhere.^12,32^

This kind of study, despite providing valuable insight, is limited to relaxed, minimal energy structures, which are only attractors of the configuration space of the highly mobile lattice of HOIHPs. Moreover, the attribution of energy differences to the HB formation is somewhat doubtful, considering that MA are cations with the positive part located at the NH_3_ group. A method for disentangling the HB energy from the Coulomb energy in HOIHPs has been shown in ref (33), reaching in halide perovskites values between 0.02 and 0.27 eV per organic cation.

Molecular dynamics (MD) simulation allows exploration of the configuration space, which is of particular importance regarding the rotational motion of the organic cations and the *dance* of the halide ions. Some important milestones of HOIHP MD studies include first ab initio MD (AIMD),^44,45^ the development of a classical force-field,^46,47^ and computation of phase transitions temperatures by means of machine-learning force fields (MLFF).^48^ MLFF allow very long MD simulation times, with forces computed with accuracy very close to DFT (used to train the MLFF), which is essential for modeling the intricate dynamics of HBs in MAPbBr_3_. Let us mention the previous studies that have focused on HBs in MAPbBr_3_. Saleh et al.^49^ performed both AIMD and classical MD, obtaining statistical descriptors of time and space correlation of MA orientation, polarization, and its influence on local electronic structure. They concluded that the HBs control the energetics of MA orientations. Maity, Verma, Ramaniah, and Srinivasan^31^ have performed an ab initio molecular dynamics study for temperatures of 40, 180, and 300 K, representative of each crystalline phase of MAPbBr_3_, obtaining several properties related to crystal structure, absence of ferroelectricity, the orientation dynamics of the cations, and lifetimes of HBs.

In a recent ab initio molecular dynamics (AIMD) study,^39^ the geometric conditions for the existence of HBs at the molecular level were defined. However, these were available only for a temperature of 350 K. In this work, we present new MD simulations performed with an MLFF.^50^ MLFF facilitates the study of hydrogen bond dynamics over longer time scales, allowing for a detailed understanding of the activation energies associated with bond dissociation. The animation videos V1–V6 in the Supporting Information show the dynamics of HBs for different temperatures. The bonds tend to break due to the translation of the methylammonium (MA) cation and the change in orientation of the C–N vector. To a lesser extent, this occurs due to rotation of the CH_3_ or NH_3_ groups. Generally, when a bond breaks, it does not reform but rather establishes a new bond with another Br. At low temperature (125 K), the ammonium group forms three N–H···Br bonds most of the time. These bonds frequently involve two Br atoms on opposite edges of the same face and another Br on an edge of another face. When a bond breaks, another bond quickly forms with a different Br, typically associated with a slight change in the orientation of the C–N vector or a rotation of the ammonium group around this vector. None of the observed MA molecules flip over during this time interval. At high temperature (325 K), the movements are broader, particularly the translation motion of the MA with large amplitude, superimposed with rotations around the C–N axis and changes in orientation. The breaking of hydrogen bonds (HBs) seems to be associated with the translational movement of the MA. The rotations are broader, but the cations remain relatively stable, and during the 2 ps of this animation, only one MA is seen to flip over. Observing the animations at intermediate temperatures, we can affirm that the bonds become more stable as the temperature decreases, particularly below 270 K, although at no temperature are the bonds totally stable. To quantify these effects, in this article, we conduct a statistical study of the lifetimes and other aspects of the HBs.

Based on these molecular dynamics simulations, we have extended the previous statistical study of HBs in MAPbBr_3_ to a wide temperature range, i.e., from 50 to 350 K. The statistical functions related to HBs here used include combined (radial and angular) distribution functions, autocorrelation functions, lifetimes (LTs), and frequency distributions. The availability of LTs as functions of temperature allows to explore Arrhenius-type behavior and to obtain activation energies associated with HB breaking.

## 2 Methods

### 2.1 MLFF Molecular Dynamics

The interatomic forces were computed using the machine-learning force fields^48,51,52^ based on atom-centered radial and angular descriptors as implemented in VASP.^53^ The force field was trained following the workflow proposed by Liang et al.^50^ Training data was collected with separate MD calculations with on-the-fly learning mode enabled, starting from 100, 160, 210, and 350 K, respectively. In these MD calculations, an isothermal–isobaric ensemble with atmospheric pressure was first adopted, with a 2 × 2 × 2 supercell and a time step of 0.5 fs. The equilibrated atomic positions and cell sizes were then applied to a second isothermal-isochoric ensemble using the same settings, where the DFT frames were collected. Langevin thermostat is adopted for both MD calculations. For the DFT calculations in the on-the-fly training, r^2^SCAN exchange–correlation functional^54^ was selected, the plane-wave basis set cutoff energy is 500 eV, and the electronic convergence threshold is 10^–5^ eV. A general force field was then trained on the collected DFT frames from the four temperatures. The detailed force field training procedures can be found in ref (50).

The MD simulations intended to obtain the HB statistics have been performed in the canonical ensemble with the Nosé thermostat,^55^ using the force field trained from all of the collected DFT frames as explained in the previous paragraph. The simulation cell contains 2592 atoms, which corresponds to a 6 × 6 × 6 supercell of the cubic phase primitive cell. The dimensions of the simulation cell for each temperature were determined as the average values in a previous simulation with an isothermal–isobaric ensemble. The equations of motion were integrated with a time step of 0.5 fs, while the coordinates were saved every two time steps. The equations of motion need to be integrated with such small timesteps to obtain accurate vibrational frequencies.^56^

### 2.2 Hydrogen Bonding Analysis

**Figure 2:**
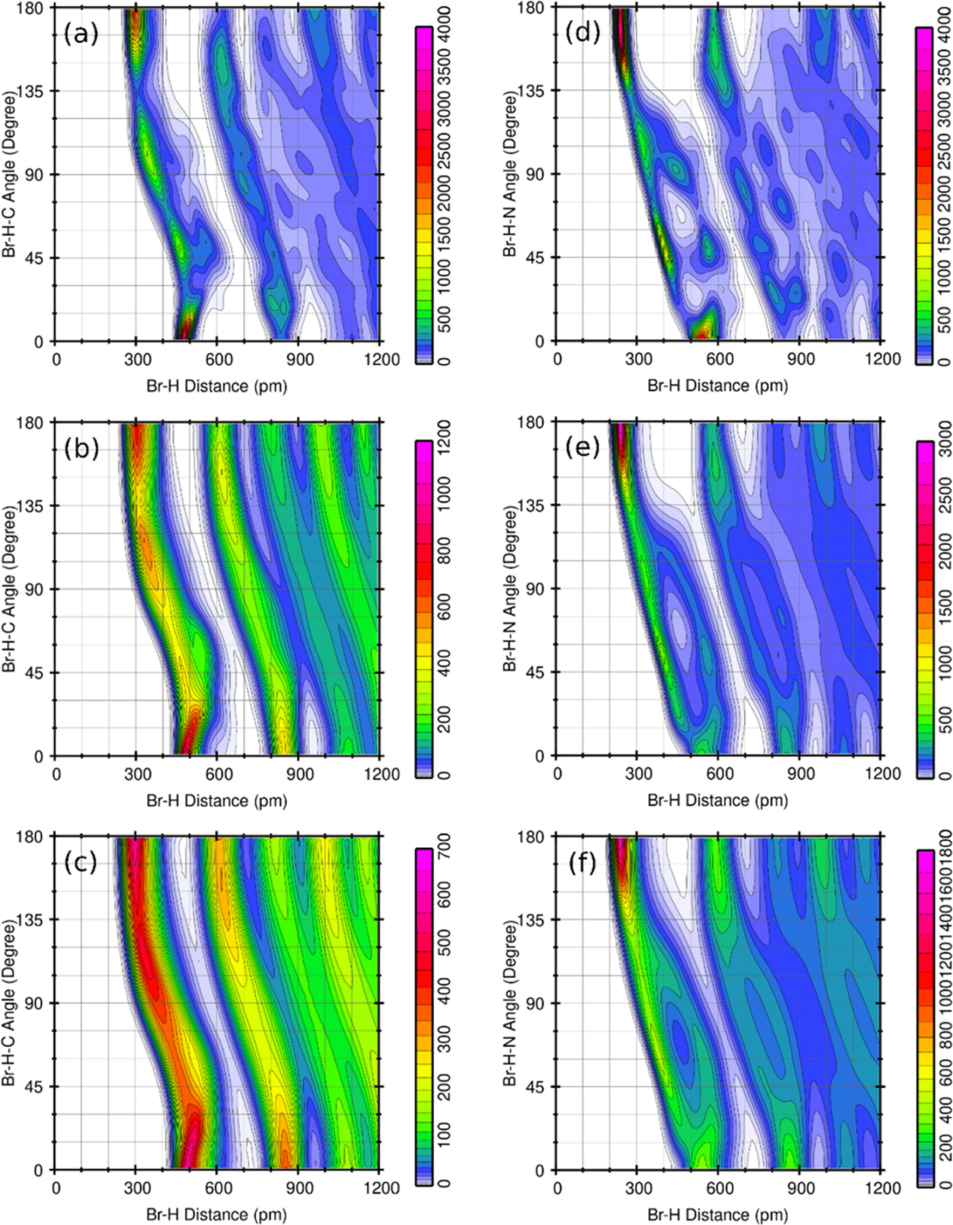
Combined distribution functions of Br–H distance in pm (horizontal axes) with ∠(Br–H-X) angle (X = C or N) in degrees (vertical axes) for temperatures at 70 K (a and d), 175 K (b and e), and 350 K (c and f). The color scale indicates the configuration frequency for each of the 100 × 100 bins, which is proportional to the configuration probability.

To characterize the X–H···Br HB (X = C or N), we have used a combined distribution function (CDF) of the distance H–Y and the angle Br–H–X. This CDF describes the joint probability of finding a hydrogen-acceptor distance, simultaneously with a certain angle Br–H–X, in the ensemble generated from the MD. These CDFs have been computed using the TRAVIS code.^57^Figure 2 shows the CDF with the HB length plotted on the horizontal axis, while the angle of the HB is displayed on the vertical axis. The red spots indicated the ranges of maximal probability to find the distance H–Br and the angle Br–H–X in the ensemble generated by molecular dynamics.

The two-dimensional (2D) contour plots in Figure 2 show zones of high concentrations (red and purple colors), indicating the presence of HBs, when the Br–H distances are shorter than 400 pm, and the angles are close to 180°. These graphs provide information on the geometric characteristics necessary for the formation of the HB. This demonstrates a correlation between both variables, information that standard one-dimensional (1D) histograms cannot provide. In Garrote-Márquez et al.,^39^ it was shown, based on the CDFs, that instantaneous N–H···Y HBs (Y = Br or I) in the MD ensemble are characterized by simultaneous fulfilment of the conditions *d*(N–H) < 3 Å, and 135° < ∠(I–H–N) < 180°. Furthermore, C–H···Y HBs are revealed by modifying the distance condition to *d*(C–H) < 4 Å. This will be termed *the HB region* in the CDFs. The analysis of the CDFs here presented suggests that these geometric conditions remain valid for all temperatures smaller than 350 K, which, the previous study, was performed for.

The plots of Figure 2 correspond to the three phases of MAPbBr_3_: orthorhombic at low temperatures (a and d), tetragonal at intermediate temperatures (b and e), and cubic at high temperatures (c and f). It can be observed that the distributions of Br–H–C and Br–H–N angles at the HB region at 70 K attain similar values, but for 175 and 350 K, the Br–H–C angle distribution spills out of the HB region (see the expansion of the red spots and the color scales). This suggests that C–H···Br HBs are weaker than N–H···Br HBs, and additional evidence is discussed in the rest of the article. The smaller N–H distance compared to the C–H distance in the HB region may also indicate stronger N–H···Br HB, although this also aided by the positive charge of the NH_3_ group.

On the other hand, to determine the LTs of HBs, it is not sufficient to consider just a single trajectory, as a molecular dynamics simulation has chaotic behavior. The approach would be to perform a simulation where many HBs have formed and broken a significant number of times, and then obtain the time correlation function.^58^
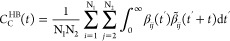
1where β_*ij*_(*t*′) = 1 if at instant *t*′ there is a HB between atoms *i* and *j* (one halide and one hydrogen, and implicitly C or N as discussed above) or zero otherwise. The function β̃_*ij*_(*t*′ + *t*) is zero if the HB breaks at any instant between *t*′ and *t*′ + *t*, or one if the HB keeps formed all of the time in this interval. The LT of the HB can be obtained as
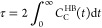
2The TRAVIS code fits the correlation function *C*_C_^HB^(*t*) to a sum of exponential functions using a least-squares procedure^57^ and performs the integration analytically. Experience indicates that to obtain converged values of lifetimes, the MD simulation time should be larger than 10τ. This means that the dynamics for low temperatures require longer simulation times. All of these calculations have been performed for temperatures ranging from 70 to 350 K, which includes the three crystal phases of MAPbBr_3_. The sampling time has been 40 ps for *T* > 125 K and 60 ps for *T* ≤ 125 K.

To obtain the activation energies associated with HB breaking, we have fitted the inverse of lifetimes with the Arrhenius equation and the Eyring equation.

We have computed power spectra as Fast Fourier transforms of velocity autocorrelation function (ACF), as implemented in TRAVIS, using the trajectory coordinates saved every one picosecond. The atomic velocities were obtained as finite difference derivatives of the atomic positions in consecutive saved frames. We used a correlation depth of 2.048 ps for the ACF, and the Verlet integrator frequency correction was used for the resulting power spectra.

In order to make a direct comparison of the resulting power spectra among different simulated temperatures, their intensities were rescaled by a factor, constraining the resulting integral of each power spectra to be equal to the number of normal modes in the system.^57^

## 3 Results

### 3.1 Verification of Geometrical Condition from the CDF

Figure 2 depicts the prevalence of HBs using CDFs, as functions of the Br–H distance and Br–H–X angle (X = C or N). The highest values are found in the purple-shaded regions, as indicated in the scale. It is observed that at higher temperatures, the red-purple-shaded areas expand, initially suggesting that the existence of HBs is greater at elevated temperatures. However, the scale also changes as the temperature increases, decreasing the maximum count values from 4000 at 70 K to 700 at 350 K. Due to this circumstance, a direct comparison of CDFs at different temperatures cannot be made. Nevertheless, it does serve to confirm the geometric conditions in which the existence of HBs is more likely. The regions of higher count can be determined by the simultaneous conditions of *d* < 3 Å, and 135° < ∠(Br–H–N) < 180° when X = N, and of *d* < 4 Å, and 135° < ∠(Br–H–C) < 180° when X = C.

### 3.2 Time Correlation Functions

**Figure 3:**
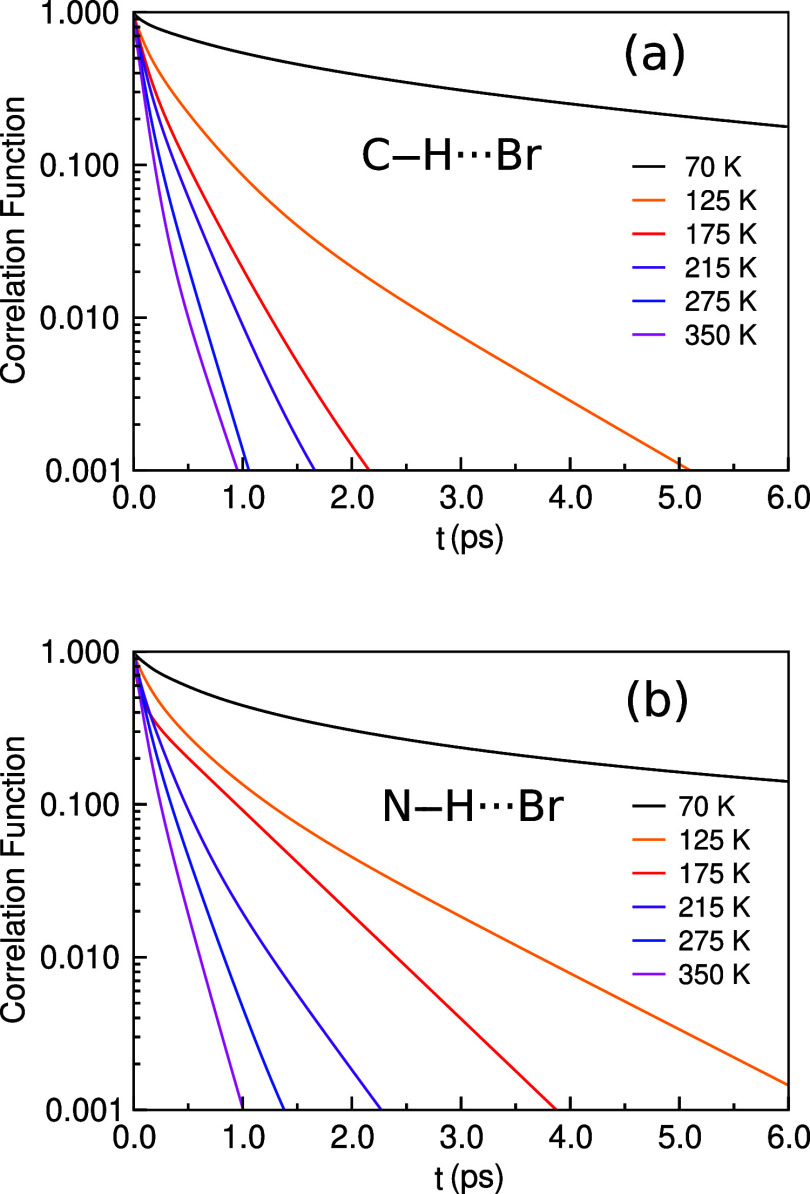
Autocorrelation functions of C–H···Br (a) and N–H···Br (b) HBs for the orthorhombic (70 K), tetragonal (125, 175, 215 K) and cubic (275 and 350 K) phases.

Once the geometric conditions that define the HBs have been confirmed to be valid for the full range of temperatures, the HB dynamics can be characterized using the time autocorrelation functions defined in eq 1, as well as the LT defined by eq 2. Figure 3 shows the correlation functions, which decay faster as the temperature increases, as expected. It can be appreciated, thanks to the logarithmic vertical scale, that none of the correlation functions decay as a single exponential function. The decay is multiexponential, and in all of the studied cases, they are very well fitted using either three or four exponential functions. The LT derived from the HBs is shown in Table 1. The number of exponential functions used in each case is also shown in Table 1. For all cases, the goodness of fit parameter *R* > 0.99997.

**Table 1:** Lifetimes at C–H···Br and N–H···Br HBs Derived from the HB Continuous Time Correlation Functions^a^

	C–H···Br		N–H···Br	
*T* (K)	τ (ps)	*N*_*f*_	τ (ps)	*N*_*f*_
70.0	7.6408	4	6.6992	4
90.0	3.4374	4	2.7417	4
100.0	2.5533	4	2.0081	4
110.0	1.5975	4	1.4053	4
125.0	0.7176	4	0.9616	4
150.0	0.5085	3	0.6800	4
175.0	0.3878	4	0.5150	3
200.0	0.3409	4	0.4166	4
215.0	0.3044	4	0.3830	3
235.0	0.2464	4	0.3383	4
250.0	0.2349	3	0.3188	4
275.0	0.2082	3	0.2787	4
300.0	0.1933	3	0.2532	4
325.0	0.1774	3	0.2267	3
350.0	0.1635	3	0.2035	4

a The number of exponential functions *N*_*f*_ is given.

It is observed that the LTs increase as the temperature decreases. Furthermore, the LTs of N–H···Br bonds are longer than the LTs of C–H···Br bonds in the cubic and tetragonal phases. However, in the orthorhombic phase of the LTs, the opposite is observed. Nevertheless, this latter comparison must be carefully evaluated, as the comparison of the LTs of C–H···Br and N–H···Br is biased due to the difference in the distance cutoffs. Hence, this comparison can be regarded only as a trend. Let of note that for the LT to be accurately computed, the TRAVIS code recommends that the MD simulation must be ten times longer than the LT. We have verified that this condition is necessary. For *T* = 125 K, a sampling time much larger than 10τ, 60 ps, was needed to obtain a converged value of the LT. This anomaly is probably related to the tetragonal/orthorhombic phase transition. The LTs for 70 K are slightly larger than one-tenth of the simulation time, but they still fit into the Arrhenius trend, as is discussed in the next section. For 50 K, LTs of 16 and 18 ps were obtained for N–H···Br and C–H···Br bonds, respectively, but these values seem inaccurate because they fall well below the Arrhenius trend and are also much larger than 10% of the simulation time. Extending the simulation time to obtain accurate LTs for 50 K would have required excessive storage of atomic coordinates; hence, it was not done.

### 3.3 Arrhenius Plots

**Figure 4:**
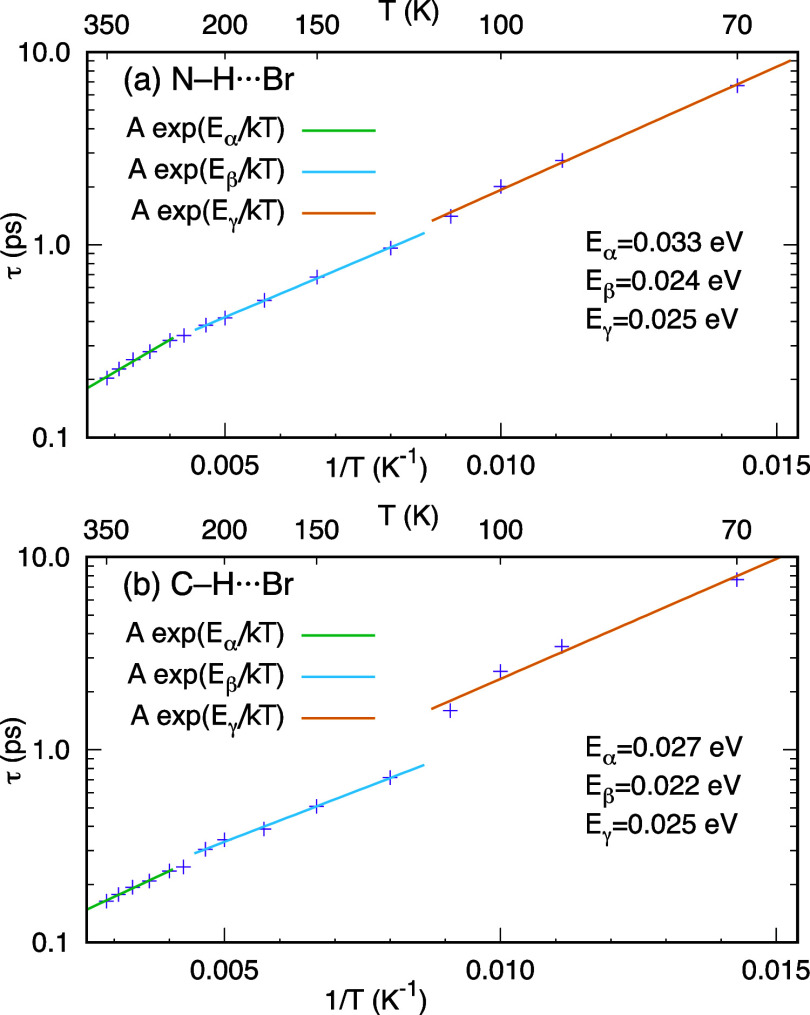
Arrhenius equation plot between the lifetimes of N–H···Br and C–H···Br hydrogen bonds and the inverse temperature for MAPbBr_3_.

Figure 4a depicts the relationship between the LT of N–H···Br HBs and the inverse temperature. The computed LTs, marked with purple crosses, suggest a linear dependency when plotted on a logarithmic scale of LTs against the inverse of temperature, which is characteristic of Arrhenius equation plots. The Arrhenius-type behavior of LTs refers to the temperature dependence of the reaction rate of HB breaking. According to the Arrhenius equation,^59,60^ the reaction rate *k* can be expressed as
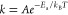
where *A* is a constant pre-exponential factor, *E*_a_ is the activation energy of the reaction, *k*_B_ is the Boltzmann constant, and *T* is the absolute temperature. In our context, the reaction is the breaking of the HB, and the reaction rate is the inverse of the lifetime *k* = 1/τ. Hence, the activation energy can be obtained from the least-squares fit of the linearized equation of ln τ vs 1/*T*
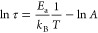
The slope of the fitted line, multiplied by *k*_B_, provides the activation energy for the dissociation of the HBs. The different activation energy values for three distinct phases, α (cubic), β (tetragonal), and γ (orthorhombic), denoted by *E*_α_, *E*_β_, and *E*_γ_, respectively, reveal subtle differences in the stability of bonds under different phase conditions. Notably, the α phase exhibits a slightly higher activation energy than the β and γ phases. Nonetheless, the linear trend is clear for the three phases, and the correlation coefficients are 0.994, 0.999, 0.996 for the α, β, and γ phases, respectively. There is one notable outlier from the linear trend, which has been dismissed from the linear fits. At 235 K, MAPbBr_3_ is in the α phase but close to the α–β transition. The N–H···Br bond lifetime at 235 K is smaller than it should be, and it seems to fit better with the line of the β phase. This anomaly may be related to the fact that structural parameters show a phase with mixed cubic-tetragonal character.^50^

A similar analysis for C–H···Br HBs is shown in Figure 4b. Comparatively, the slopes of the lines indicate different activation energies for C–H···Br and N–H···Br HBs. Figure 4 indicates a slightly lower activation energy, for the cubic and tetragonal phases, for C–H···Br than for N–H···Br bonds, consistent with the idea that N–H···Br HBs are stronger. Moreover, the lifetime of C–H···Br HBs shows the same kind of deviations at 235 K that was mentioned for N–H···Br bonds. Another anomaly appreciated in Figure 4b is a marked jump of the C–H···Br bond LT between temperatures 125 and 110 K, coincident with the transition between the tetragonal and orthorhombic phases. This drop marks the change from higher LTs of C–H···Br bonds to higher LTs of N–H···Br bonds.

The variation of activation energies between the different phases is somehow related to the changes in distribution of the organic cation orientation (see Figure 6 in ref (50).) and the differences in the pattern of octahedral tilting between the three phases. In the orthorhombic phase, the MA cations have their C–N axis confined almost parallel to the ab plane, while the azimuthal angle is concentrated in small ranges near six preferred values. In contrast, in the tetragonal phase, the C–N axis gets values around ±31.4° with the *ab* plane, with four preferred azimuthal angles. The cubic phase also displays a different pattern; the C–N axis orientation shows a broad distribution with minima in the directions toward the nearest Pb atoms. Liang et al.^50^ also showed that the spatial correlations of the MA orientations directly reflect the crystal symmetry. The octahedral tilting also leads to different environments for MA cations, modifying the number of halide atoms within bonding distance and the total energy.^41,42^ The differences in the distribution of orientations between the different phases are so marked that the variations in activation energies appear rather small.

The Eyring equation^61^ fits the data worse than the Arrhenius equation, confirming that the exponential prefactor is better described as temperature independent. The Eyring-type plots in the Supporting Information show visually important deviations from linearity, and the correlation coefficients are smaller than those in the Arrhenius case. The inadequacy of the Eyring equation for HB breaking can be understood as the Eyring equation was derived for bimolecular reactions of the type *A* + *B* → *X*^‡^ → *C*, where *A* and *B* are the reactants, *X*^‡^ is a transition state, and *C* is the product.^62^

### 3.4 Neighbor Analysis

**Figure 5:**
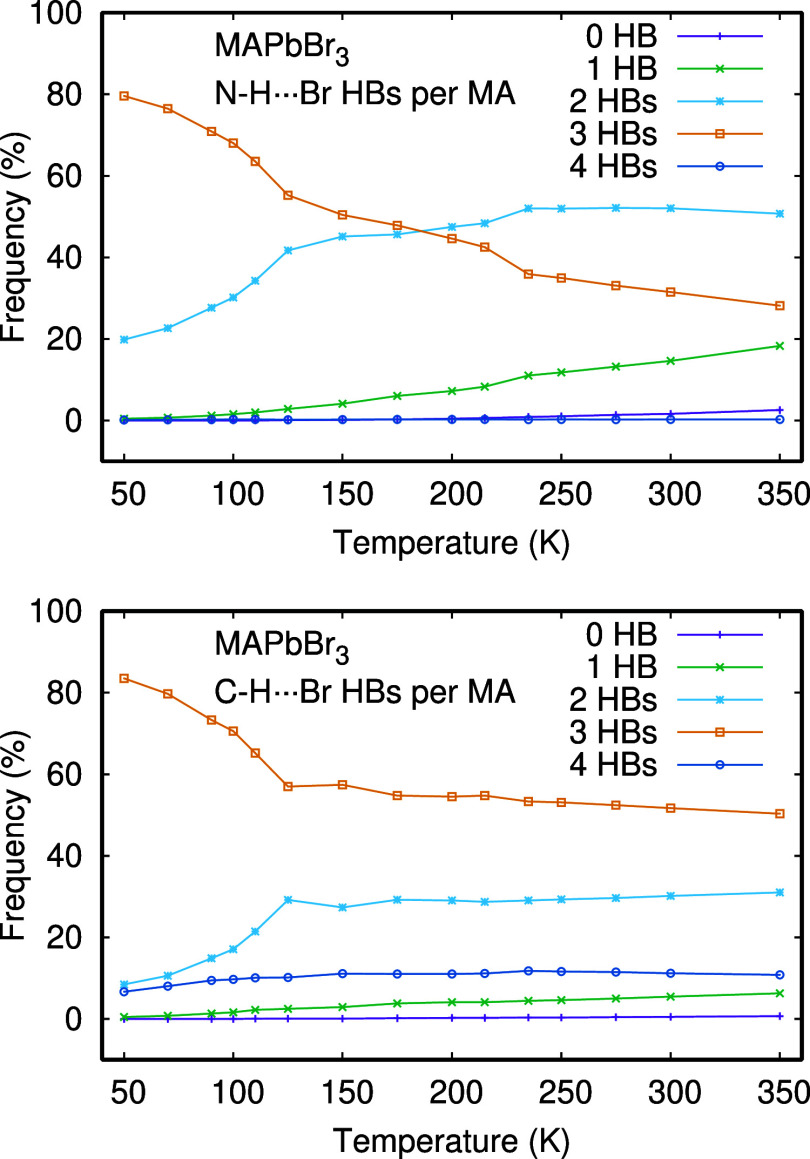
Distribution of the number of bromine ions linked to MA cations through N–H···Br or C–H···Br hydrogen bonds, as a function of temperature.

Figure 5 shows, as a function of the temperature, the statistical distribution of the number of bromine ions linked to MA cations through N–H···Br or C–H···Br HBs. These numbers correspond to the number of HB per MA cation, disregarding the very rare configurations where some Br establish more than one HB with the same cation (none has been seen in the animations). At low temperatures, MA cations establish N–H···Br HBs with three Br (∼80% of configurations at 50 K) or two Br (20% at 50 K), while other coordination numbers are negligible. At high temperatures, 2-fold coordination overcomes 3-fold coordination (51 vs 28% at 350 K). The crossover between 3-fold and 2-fold coordination through N–H···Br HBs takes place near 175 K. Moreover, there is a pronounced change in slope at 125 K, coincident with the orthorhombic/tetragonal phase transition. A similar, although less pronounced change, takes place near the tetragonal/cubic phase transition, at 235 K. The likelihood of MA forming just one HB is negligible at low temperature, but it begins to rise after 125 K, reaching 18% at 350 K. The statistics of C–H···Br HBs has similarities and differences. Three-fold coordination is the most frequent one in the full temperature range. Two-fold coordination is the second in importance. Like for N–H···Br HBs, there is a pronounced change in slope around 125 K. Surprisingly, 4-fold coordination is the third most frequent case, with a 10% share. We think that this abundance of high coordination is a consequence of the enlarged C–H···Br distance cutoff compared with the N–H···Br distance cutoff, and the confinement of MA in the cuboctahedra cages. The N–H···Br bonds are stronger than the C–H···Br ones, as indicated by shorter bond distances and the effect on the power spectra discussed below. This can be summarized as follows:

- HBs in N–H···Br are potentially stronger but are more impacted by rising temperatures.
- HBs in C–H···Br maintain a certain stability throughout the analyzed temperature range.

We could attempt to estimate the HB energies by comparison with the obtained activation energies. Under this assumption, the energy per cation can be modeled as follows.
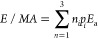
where *n*_*i*_ is the number of bonds, *p*_*i*_ is the probability (frequency/100%), and *E*_a_ is the Arrhenius activation energy. As a result, we obtain that the HB energy per cation for 250 K in the N–H···Br HB is 0.073 eV; for 150 K, it is 0.059 eV, and for 70 K, it is 0.069 eV.

A detailed calculation^33^ of the HB energy per cation of MAPbBr_3_ returned a value of 0.26 eV. This calculation is based on static relaxed structure, and the HB energy was obtained from a partition scheme of the electrostatic energy. With our molecular dynamics Arrhenius approach, we have obtained 0.069 eV for 70 K and 0.075 eV for an ideal structure with 100% of cations with three HBs. Our values are about one-third/fourth of the former values, which is understandable due to the differences of the calculation approach, letting aside the difficulty in identifying activation energies with HB energies.

Anyway, these activation energies are located at the bottom of the table of typical of HBs energies (0.009–1.7 eV).^63^ The proximity of these energies to thermal energy suggests that the effect of HBs in stabilizing halide perovskites is rather small.

On the other hand, HB energy is defined a dissociation of the acceptor and donor.^63^ The dissociation limit is not reached in the dynamics of the condensed phase. As mentioned in Section 1, the animations show that when a HB is broken, a new HB forms with a nearby bromide in a short time. Therefore, the activation energies could be related to the transition path between two HB configuration. Hence, the dissociation HB energies are, in principle, higher than activation energies.

### 3.5 Vibrational Power Spectra

**Figure 6:**
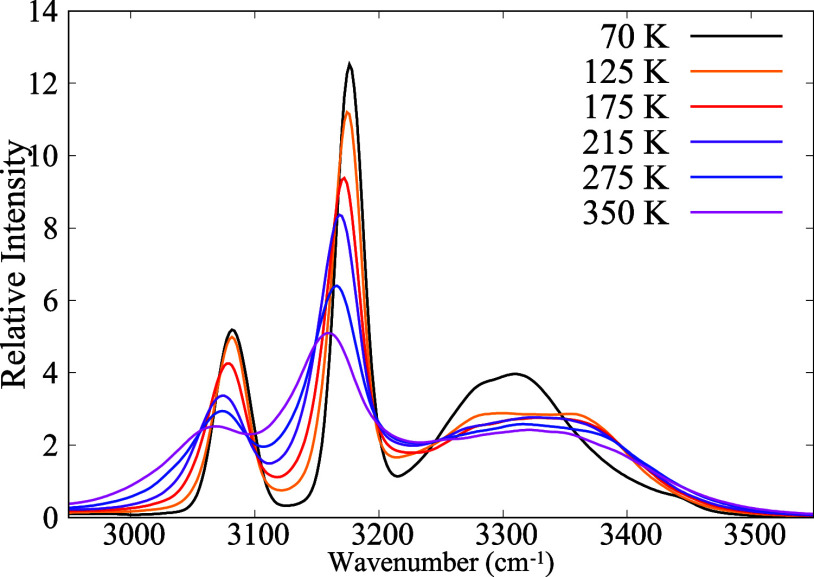
Power spectrum in the region of N–H and C–H bond stretching as a function of temperature.

Figure 6 shows the power spectrum for selected temperatures in the range of wavenumbers that encompass the N–H and C–H bond stretching. The power spectrum provides a vibrational density of states that includes anharmonic effects.^56^ Its interpretation is aided by knowledge of the harmonic normal modes of an isolated MA^+^ cation, as shown in Table 2. These modes have been computed ab initio with the same functional employed to generate the MLFF.^50^ Modes 1 and 2 are degenerate with E symmetry, and the same is true for modes 4 and 5. The small splitting observed in Table 2 is a consequence of the breaking of rotational symmetry in the calculations with the periodic boundary conditions.

**Table 2:** Normal Modes of the N–H and C–H Bonds Stretching in the Isolated MA^+^ Cation

mode	wavenumber (cm^–1^)	symmetry	description
1	3491	E	NH_3_ asym. stretching
2	3489		
3	3413	A_1_	NH_3_ sym. stretching
4	3199	E	CH_3_ asym. stretching
5	3198		
6	3090	A_1_	CH_3_ sym. stretching

Comparing the curves of Figure 6 with the wavenumbers in Table 2, we assign the two narrow peaks at the left side to the CH_3_ symmetric and asymmetric stretching. The isolated cation normal-mode frequencies are modified by interaction with the environment and anharmonicities. The frequencies of these modes (peak maximums) in the perovskite environment vary from 3083 to 3061 cm^–1^ and 3179 to 3158 cm^–1^ when temperature raises from 50 to 350 K. We conclude that normal modes of C–H bond stretching are weakly affected by the environment and anharmonicity. In contrast, the higher frequency modes, associated with the N–H bond stretching, are strongly broadened and red-shifted in comparison with the isolated cation modes. This is a signature of the HBs. The shape of this band is rather constant for the cubic and tetragonal phases but changes noticeably for temperatures below 125 K, in the orthorhombic phase.

## 4 Discussion and Conclusions

In this study, it has been demonstrated that the lifetimes of the HBs in MAPbBr_3_ are in the picosecond regime, showing that greater thermal energy facilitates overcoming the energy barrier for the dissociation of the HBs. This is manifested in a decrease in the lifetimes of the HBs with an increase in temperature, following Arrhenius behavior. The specific values of the activation energy, *E*_a_, for different phases and types of HBs provide a quantitative understanding of how these factors affect the stability of the HBs. These differences in *E*_a_ between the crystallographic phases (orthorhombic, tetragonal, and cubic) and the types of HBs (N–H···Br vs C–H···Br) underline the importance of structural composition and bonding chemistry in determining the thermal stability of hydrogen bonds in MAPbBr_3_ perovskite.

The differences in the behavior of HBs between N–H···Br and C–H···Br can be attributed to two fundamental reasons related to the chemical and structural nature of the HBs formed by nitrogen and carbon, respectively.

- Nitrogen is more electronegative than carbon, meaning that N–H bonds are more polarized than C–H bonds. This greater polarization of the N–H bond allows for a stronger electrostatic attraction between the hydrogen (partially positive) and the bromine atom (partially negative) in the HB, resulting in N–H···Br hydrogen bonds generally being stronger than C–H···Br bonds.
- N–H···Br bonds are shorter than C–H···Br bonds since an NH_3_^+^ group has one uncompensated proton and undergoes ionic interaction with Br^–^. Shorter and stronger HBs are generally more stable and have higher dissociation energies.

Through the CDFs, it has been possible to verify that the geometric conditions that characterize these HBs can be set constant across the entire temperature range studied, i.e., *d*(Br–H) < 3 Å, and 135° < ∠(Br–H–N) < 180° for N–H···Br, and *d*(Br–H) < 4 Å, and 135° < ∠(Br–H–C) < 180° for C–H···Br. For such conditions, the decrease in the maximum count values of the HBs as the temperature increases is noteworthy. With these definitions, it turns out that the MA cations are mostly linked to Br anions by two or three N–H···Br and C–H···Br bonds. For low temperature, three HBs of each type is the most frequent case, decreasing with increasing temperature. For high temperature, two N–H···Br bonds are the most frequent case, with a crossover near 175 K. In contrast, 3-fold C–H···Br bonding is the most frequent case for all temperatures.

The power spectra show a redshift with increasing temperature for the signals associated with C–H stretching modes (∼3000–3200 cm^–1^) and for other modes with lower wavenumber. The same trend is observed for the N–H stretching modes (∼3300 cm^–1^), jointly with a strong broadening, which is a signature of HB. Although C–H···Br bonds have lifetimes and activation energies similar to those of N–H···Br bonds, they have no clear effect on the vibrational properties at any temperature. Hence, the mere existence of C–H···Br bonds is not due to chemical interaction, but it is probably due to the confinement of MA+ in the cuboctahedral cage of the perovskite structure.

## Supporting Information Available

Combined distribution functions of the H–Br distance with either H–N or H–C distance; correlation plot of distances X–H and H–Br in N–H···Br and C–H···Br bonds; power spectra; HB existence autocorrelation functions at 350 K for different simulation lengths and different values of the Nose thermostat mass; some results from the MD simulation at 350 K (energies vs simulation time) and velocity distribution function for each species; distribution of the number of bromine ions linked to MA cations through N–H···Br or C–H···Br HBs, as function of temperature; HB autocorrelation functions for different correlation depths; comparison of linear fits with the Arrhenius equation; and the Eyring equation for the N–H···Br bonds (PDF)
Animation of HB dynamics at 90 K (MP4)
Animation of HB dynamics at 125 K (MP4)
Animation of HB dynamics at 275 K (MP4)
Animation of HB dynamics at 300 K (MP4)
Animation of HB dynamics at 325 K (MP4)
Animation of HB dynamics at 350 K (MP4)
