# Supplementary material for: Picosecond Lifetimes of Hydrogen Bonds in the Halide Perovskite CH3NH3PbBr3
Source: J Phys Chem C Nanomater Interfaces. 2024 Nov 26;128(49):20947–56. doi: 10.1021/acs.jpcc.4c04686 (PMC11648085; doi:10.1021/acs.jpcc.4c04686)
Supplement: Supplementary file 1 — jp4c04686_si_001.pdf [file jp4c04686_si_001.pdf]

Supporting Information

Picosecond lifetimes of hydrogen bonds in the halide perovskite $CH_3NH_3PbBr_3$

Alejandro Garrote-Márquez,[a] Lucas Lodeiro,[b] Norge Cruz Hernández,[a] Xia Liang,[c] Aron Walsh,[c] and Eduardo Menéndez-Proupin[a*]

[a] *Departamento de Física Aplicada I, Escuela Politécnica Superior, Universidad de Sevilla, Seville E-41011, Spain*

[b] *Departamento de Química, Facultad de Ciencias, Universidad de Chile, Las Palmeras 3425, Ñuñoa 7800003, Santiago, Chile*

[c] *Thomas Young Centre and Department of Materials, Imperial College London, London SW7 2AZ, UK*

*Corresponding author. E-mail: emenendez@us.es

Combined distribution functions of X-H and H···Y (X=N and C, Y=Br) distances in $MAPbBr_3$

A little of additional information is provided by the CDFs of distances H—Br and X—H. The CDFs for temperatures 100 K and 350 K, typical of the orthorhombic and cubic phases, are shown in Figure S1. At 100 K, there is a characteristic "island" of low H—Br distance, a signature of the N–H···Br bond. That kind of "island" does not appear for the C–H···Br bond. The CDFs are broadened at 350 K, and the "island" disappears.

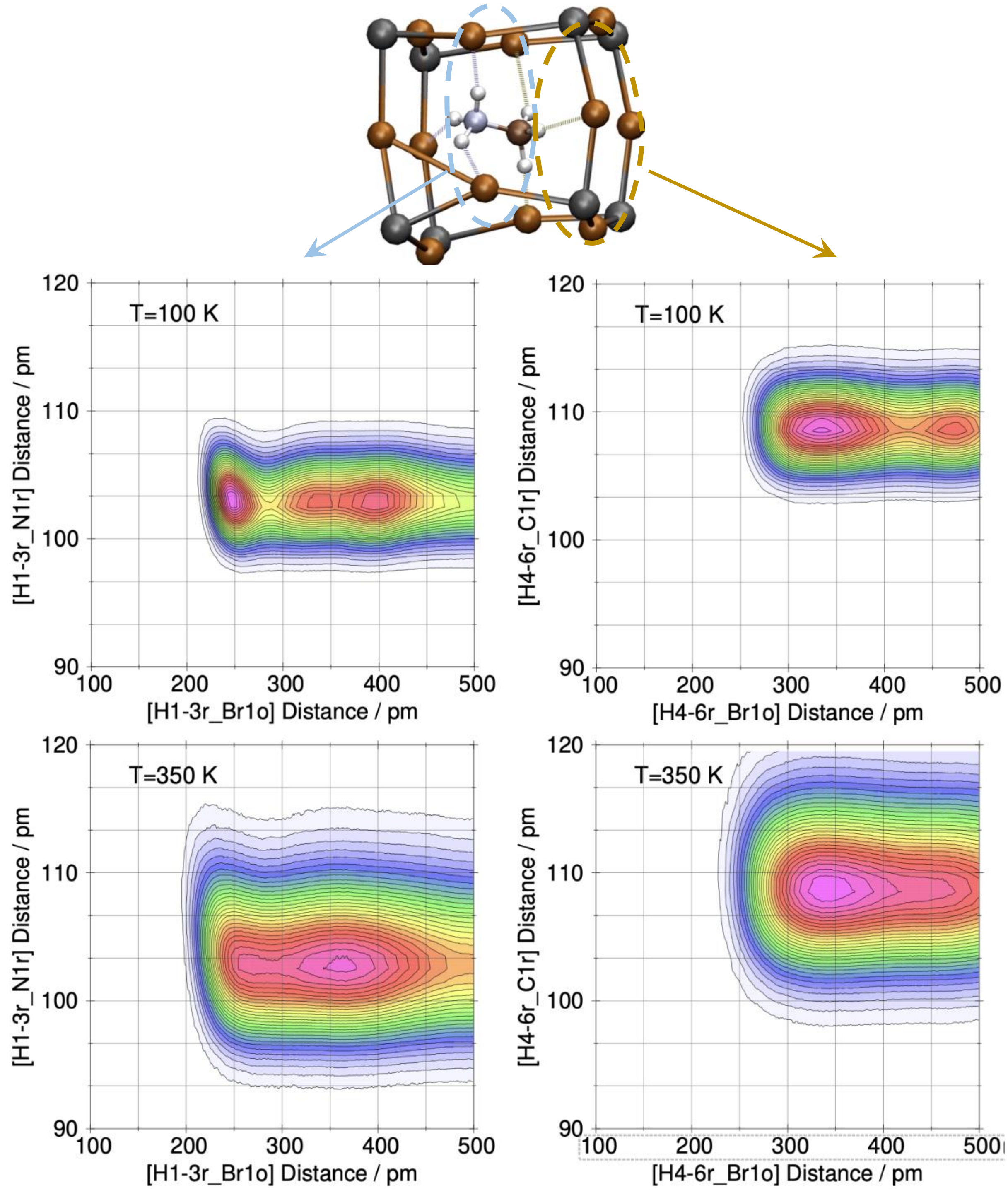

Figure S1. Combined distribution functions of the H-Br distance with either H-N or H-C distance.

Let us see the information given by the correlation functions of the same distances (see J. Chem. Phys. 152, 164105 (2020); doi: 10.1063/5.0005078). These are computed by the TRAVIS code, by subtracting from the CDFs, the Cartesian product the histograms of the two distances. Positive values indicate that the probability of finding this configuration is larger than if the two quantities would be uncorrelated, while negative values depict the opposite situation. Figure S2 shows that configurations with shortened H—Br distance and enlarged X—H distance present positive correlation. This correlation reveals both kinds of HBs at both temperatures.

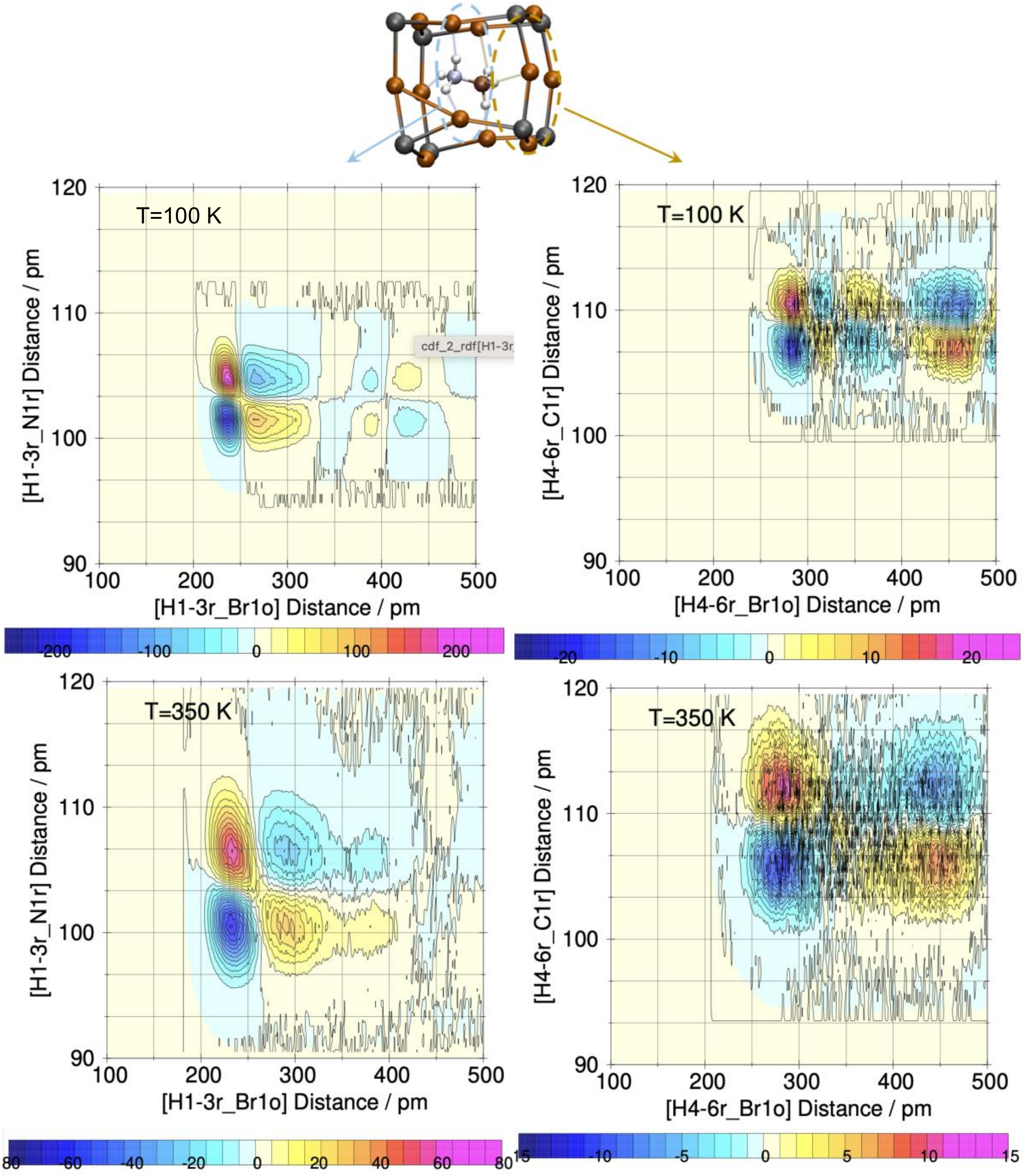

Figure S2. Correlation plot of distances X—H and H—Br in N─H···Br and C─H···Br bonds.

Power spectrum of $MAPbBr_3$ and the choice of thermostat mass (SMASS)

To ensure that the thermostat does not interfere with the dynamic of the hydrogen bonds, we have explored the effect of the Nose thermostat mass on the dynamic properties, which in the VASP code is regulated by the parameter SMASS. This parameter is automatically set in VASP so that the oscillation of the temperature has a period of 40 MD steps. In our system, that automatic value is SMASS =0.04. With the time step of 0.5 fs, the oscillation period is 20 fs, corresponding to a wavenumber of 1668 cm^{-1}. This is very close to the centre of the power spectrum of $MAPbBr_3$ (see Figure S3 below) and should be optimal for efficient thermalization, but it may interfere with the cation dynamics, and it may affect the computed lifetimes. Hence, we tried with SMASS=0.2 and SMASS=1.0, the latter corresponding with an oscillation of temperature every 100 fs or a wavenumber of 334 cm^{-1}, which is in the range of normal modes of the inorganic sublattice. As Figure S3 shows, the differences in the power spectrum are negligible.

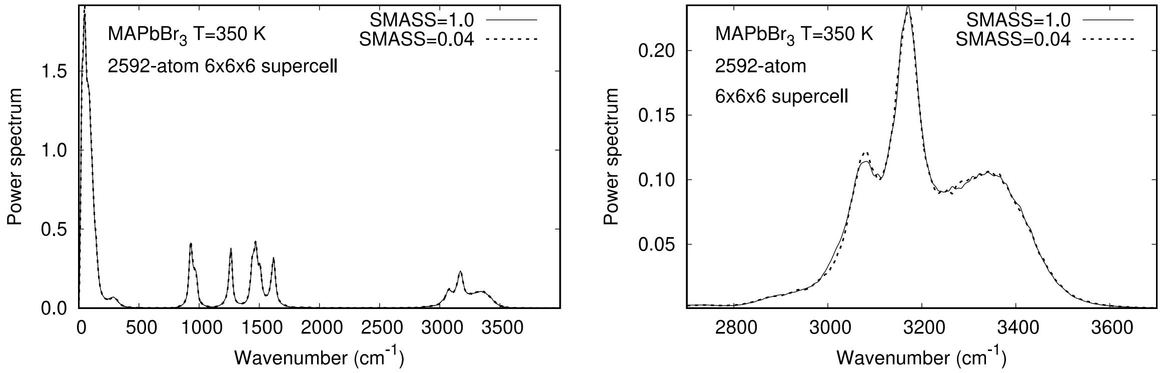

Figure S3. Power spectrum de $MAPbBr_3$. Comparison of the power spectrum for two different thermostat masses (VASP parameter SMASS).

Figure S4 shows that the N─H···Br bond existence autocorrelation function at 350 K. As before, the effect of the SMASS parameter and the simulation time is small.

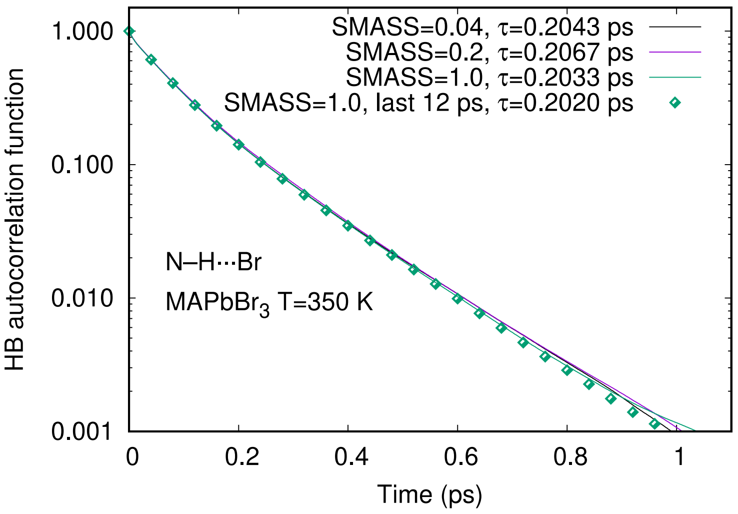

Figure S4. HB existence autocorrelation function of $MAPbBr_3$ at 350 K, computed with different thermostat mass parameters (SMASS), and with different simulation lengths (20 ps if not specified.). The HB lifetimes τ are indicated.

Figure S5 shows several indications of the quality of the MD simulation. In part (a) is the trace of the potential energy, the total energy (potential+kinetic) and the conserved quantity in the

NPT dynamics. Parts (b-f) show the velocity distribution function for each element, and the temperature obtained by fitting with the Maxwell distribution.

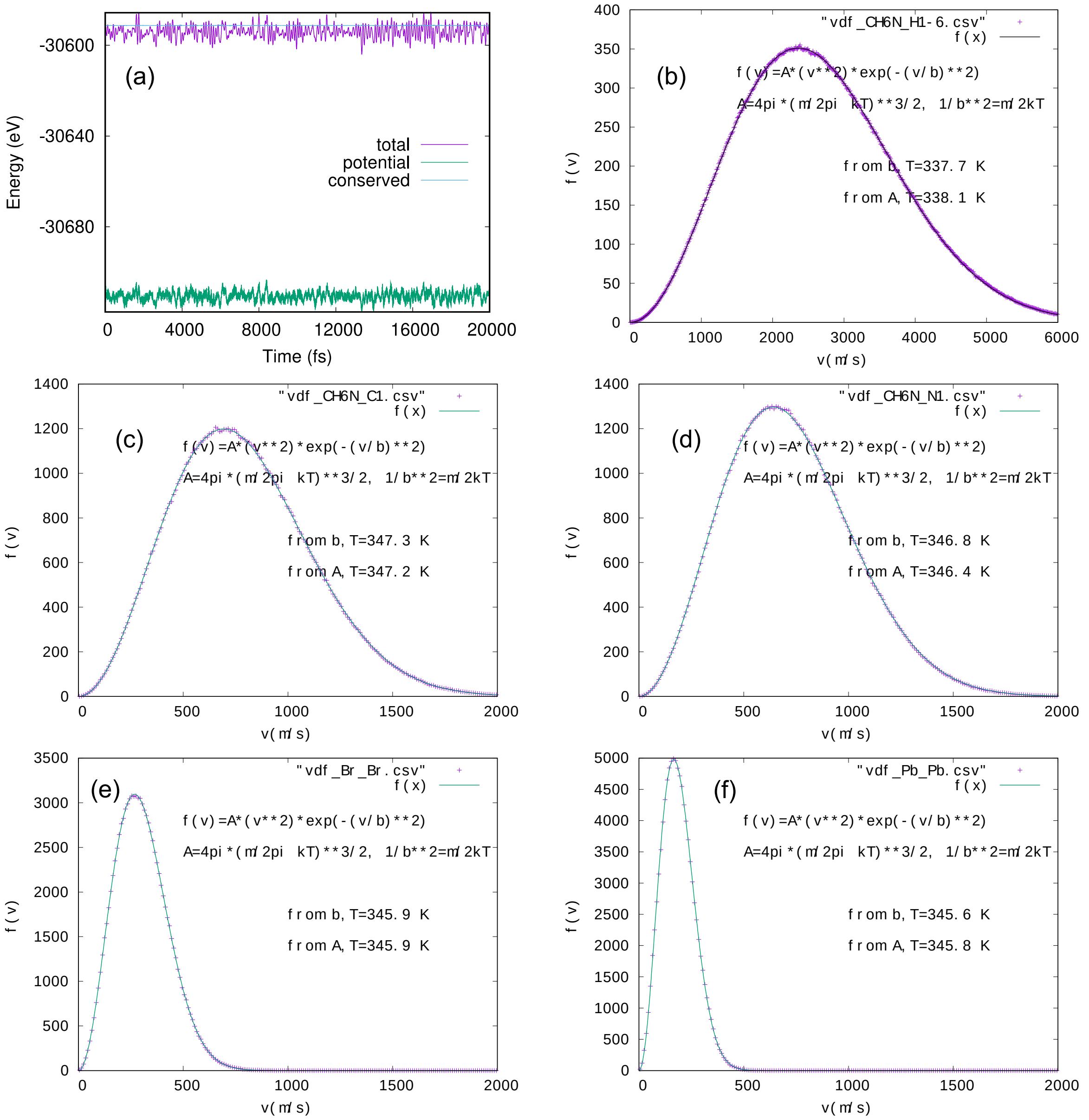

Figure S5. Some results from the MD simulation at 350 K. a) Energies along the simulation time: potential energy, total (potential+kinetic), and the conserved energy, which is the Nose Hamiltonian. b-f) velocity distribution function for each species, fits to the Maxwell distribution functions, and the temperatures derived from the standard deviation and from the pre-exponential factor.

Neighbor analysis

The following information appears in files cond*.txt and travis.log, after a DACF calculation. For example, for temperature 350 K we obtain

- 0 Neighbors: 2.5753 percent of the time (111255 hits).
- 1 Neighbors: 18.2884 percent of the time (790061 hits).
- 2 Neighbors: 50.7102 percent of the time (2190681 hits).
- 3 Neighbors: 28.1628 percent of the time (1216635 hits).
- 4 Neighbors: 0.2620 percent of the time (11318 hits).
- 5 Neighbors: 0.0012 percent of the time (50 hits).

Based on the above numbers, one can say that on average each MA links with Br through 1 HB 18.3% of the time, 2 HBs 50.7%, 3 HBs 28.2% and makes no HB 2.6% of the time. 4 HBs also appear 0.26% of the time, they can be things like an H linked to 2Br and the other 2 H to two other Br, or 2H with 2Br each, or an H with 3 Br and the other with a Br. 0.26 % is little, but there are 11318 hits, it seems like a good statistic. The same analysis was done for all other simulated temperatures, and it is summarized in Figure S6.

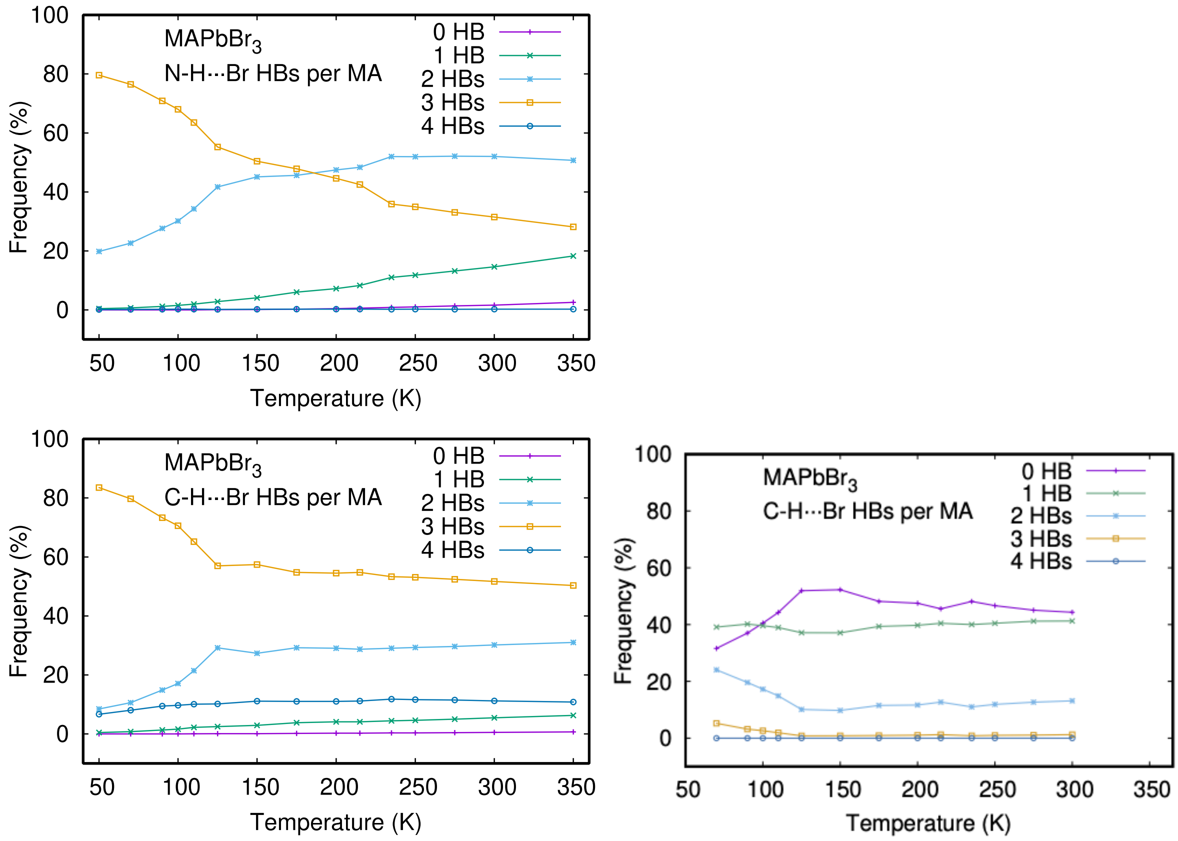

Figure S6. Distribution of the number of bromine ions linked to MA cations through N—H···Br or C—H···Br HBs, as function of temperature. At the bottom we see to versions of the C-H…Br statistics, with cutoff distance of 400 pm (left) and 300 pm (right).

For 350 K we have evaluated the effect of the SMASS Parameter. The largest variation is 0.2%.

	SMASS=1.0	SMASS=0.04
- 0 Neighbors:	2.5753%	2.4983%
- 1 Neighbors:	18.2884	18.2802%
- 2 Neighbors:	50.7102	50.5972%
- 3 Neighbors:	28.1628	28.3776%
- 4 Neighbors:	0.2620	0.2461%
- 5 Neighbors:	0.0012	0.0005%

Effect of the correlation depth on the computed lifetimes

Figure S7 shows one of the HB autocorrelation functions for different correlation depths. The correlation depth, parameter of the TRAVIS code, is the maximum time the autocorrelation function is computed for, and this affects the computed HB lifetimes, which are indicated in the Figure. It is shown that the default parameter, 18 ps for a simulation time of 60 ps, has an excellent convergence.

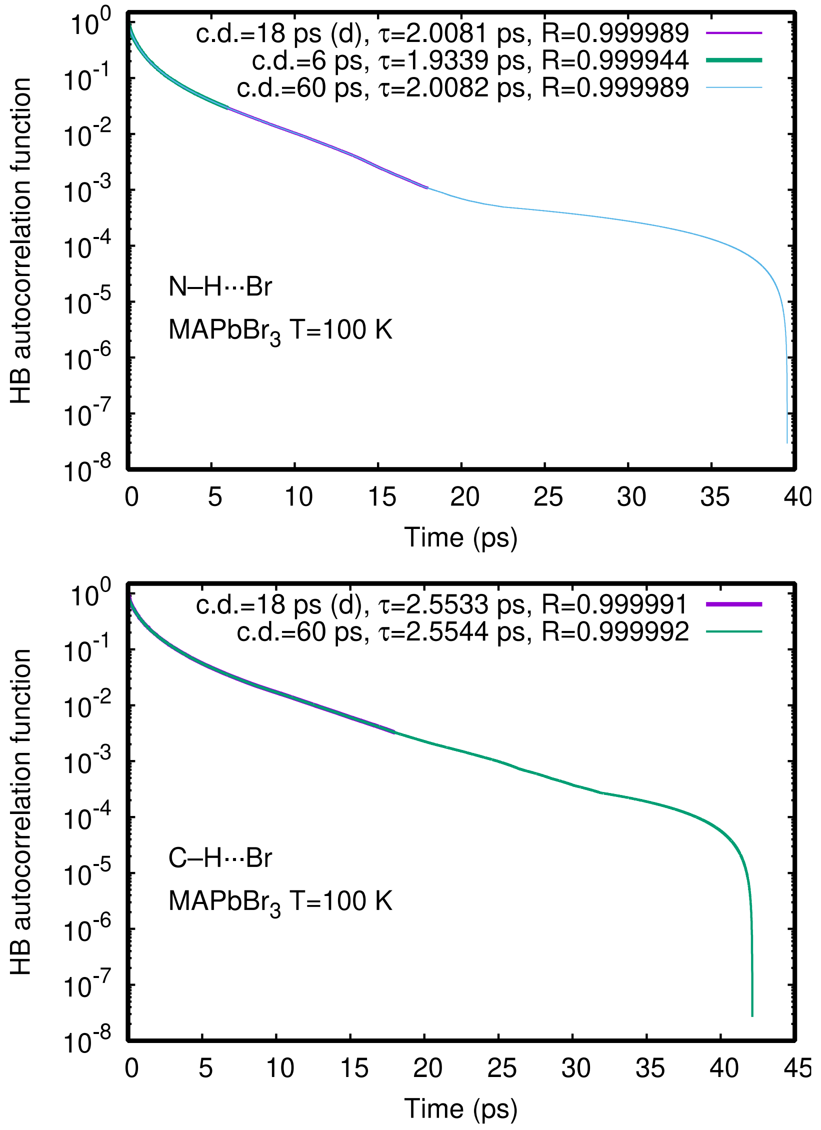

Figure S7. Effect of the correlation depth. The label (d) indicates the value suggested by the TRAVIS code.

Arrhenius vs Eyring plots

We have also analysed the LT of the HBs using the Eyring equation, checking which equation best models the behaviour of the LT of the HBs. The Eyring equation is expressed as:

$$k = \kappa \frac{k_B T}{h} e^{-\frac{\Delta g}{k_B T}}. \qquad \text{(S1)}$$

In this equation $k = 1/\tau$ represents the rate constant of the reaction. k_B is the Boltzmann constant, h is the Planck constant, Δg is the Gibbs free energy of activation per molecule. κ is a dimensionless factor called transmission coefficient. The Eyring equation can be recast as linear dependence of the inverse of temperature, as follows

$$\ln\left(\frac{1}{\tau T}\right) = -\frac{\Delta g}{k_B}\frac{1}{T} + \ln\left(\frac{k_B}{h}\right) + \ln\kappa, \tag{S2a}$$

$$\ln\left(\frac{1}{\tau T}\right) = -\frac{\Delta h}{k_B}\frac{1}{T} + \frac{\Delta s}{k_B} + \ln\left(\frac{k_B}{h}\right) + \ln\kappa, \tag{S2b}$$

where in Eq. S2b the Gibbs free energy $\Delta g = \Delta h - T\Delta s$ has been expressed by the enthalpy and entropy Δh and Δs, respectively. For Eq. S2 to be dimensionally correct, the argument of each logarithm contains implicitly a factor of dimension temperature-time, that we set as 1 K·1 ps. Hence, $\ln(k_B/h) \approx -3.8710$.

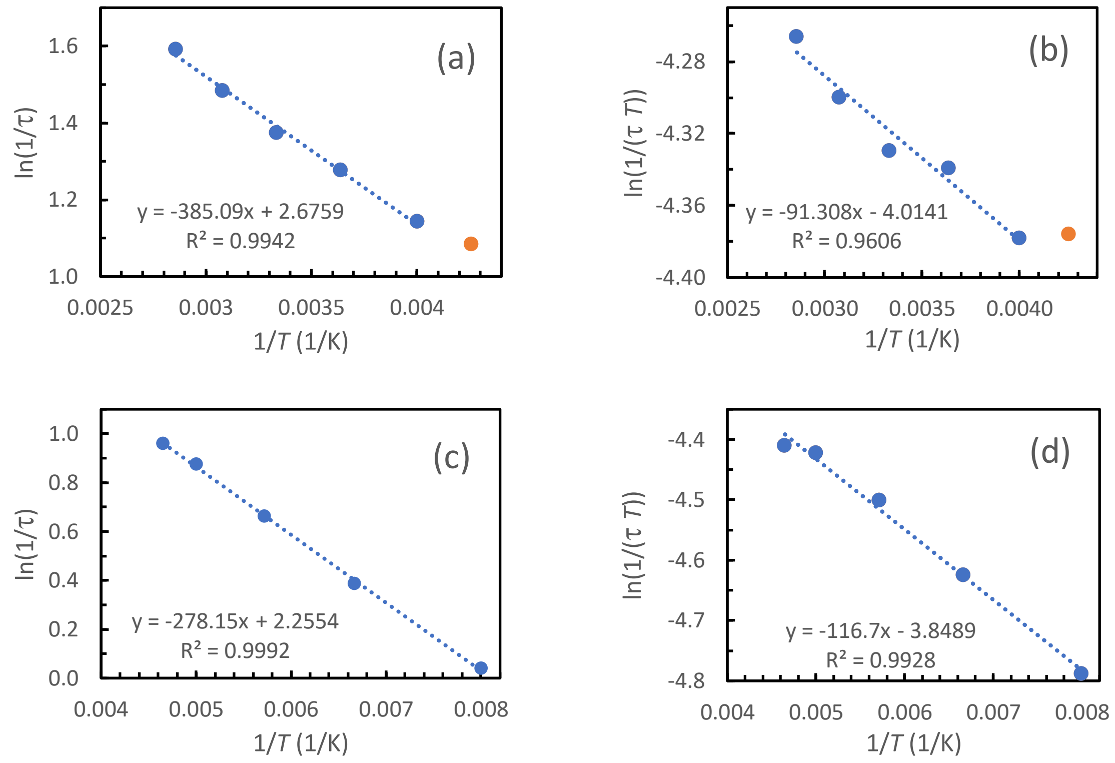

Figure S8. Comparison of linear fits with the Arrhenius (a,c) equation and the Eyring equation (b,d) for the N—H···Br bonds. The top row and bottom correspond to the cubic and tetragonal phases (c,d). The point of 235 K (in orange colour) was not included in the fit.

In case that either Δg or Δh is independent of temperature, then Eq. S2a or S2b, respectively, is linear in $1/T$. Hence, Δg or Δh can be computed from the fitted slope, while the independent term contains the transmission coefficient and the change of entropy in Eq. S2b. We have

worked with Eq. S2b, assuming that Δh is constant. Figure S8 above suggests that the linear fit of $1/\tau T$ to the Eyring equation is worse than the linear fit of $1/\tau$ to the Arrhenius equation.
